# Lapatinib-induced enhancement of mitochondrial respiration in HER2-positive SK-BR-3 cells: mechanism revealed by analysis of proteomic but not transcriptomic data

**DOI:** 10.3389/fmolb.2024.1470496

**Published:** 2024-09-30

**Authors:** Dmitry Kamashev, Nina Shaban, Galina Zakharova, Alexander Modestov, Мargarita Kamynina, Sergey Baranov, Anton Buzdin

**Affiliations:** ^1^ Endocrinology Research Center, Moscow, Russia; ^2^ Institute of Personalized Oncology, I. M. Sechenov First Moscow State Medical University, Moscow, Russia; ^3^ Shemyakin-Ovchinnikov Institute of Bioorganic Chemistry, Moscow, Russia; ^4^ Moscow Institute of Physics and Technology, Dolgoprudny, Russia; ^5^ PathoBiology Group, European Organization for Research and Treatment of Cancer (EORTC), Brussels, Belgium

**Keywords:** HER-targeted cancer therapy, human blood serum, lapatinib, squamous cell carcinoma SK-BR-3, drug resistance, tricarboxylic acid cycle TCA, cellular respiration, proteomics

## Abstract

Dual inhibitors of HER2 and EGFR, such as lapatinib, have shown significant efficacy for the therapy of HER2-positive breast cancer. Previous experiments showed that in cell cultures, the efficacy of lapatinib was significantly reduced by exposure to human serum and human epidermal growth factor (EGF). At the proteomic and transcriptomic levels, we examined the changes in the HER2-positive breast cancer cell line SK-BR-3 profiles upon treatment with lapatinib, either alone or in combination with human serum or EGF. Proteomic profiling revealed 350 differentially expressed proteins (DEPs) in response to lapatinib treatment at concentrations that induced cell growth arrest. Addition of human serum or EGF in combination with lapatinib prevented cell growth inhibition, and this combination treatment returned the expression of ∼93% of DEPs to drug-free levels for both human serum and EGF. Gene ontology enrichment and OncoboxPD pathway activation level analysis showed that lapatinib addition influenced mostly common functional processes revealed in RNA- and protein-based assays. However, a specific feature was observed at the proteome level: addition of lapatinib increased the expression of proteins associated with mitochondrial function and cellular respiration. This feature was not observed when using RNA sequencing data for the same experiments. However, it is consistent with the results of the resazurin test, which showed a 1.8-fold increase in SK-BR-3 cellular respiration upon exposure to lapatinib. Thus, we conclude that enhanced cellular respiration is a novel additional mechanism of action of lapatinib on HER2-positive cancer cells.

## 1 Introduction

The ERBB/HER receptor family consists of four structurally related receptor tyrosine kinases (RTKs) that regulate proliferative cell signaling and play pivotal roles in both normal physiology and proliferative diseases like cancer (Mitsudomi and Yatabe, 2010). In human, these are HER1-4: epidermal growth factor receptor (EGFR/ErbB1/HER1), ErbB2/HER2 (neu), ErbB3/HER2, and ErbB4/HER4 proteins (Arienti et al., 2019; Holbro and Hynes, 2004; Iqbal and Iqbal, 2014; Olayioye et al., 2000; Roskoski, 2014). Several growth factors, called HER ligands, have the ability to bind these receptors and initiate their activation (Komurasaki et al., 2002; Miricescu et al., 2020; Yamaoka et al., 2018). Ligand binding induces the formation of homo- and heterodimers by the ERBB/HER receptors and activates their internal kinase domain, leading to the cross-phosphorylation of the tyrosine residues in the cytoplasmic tail (Mitsudomi and Yatabe, 2010). The ligand-activated ERBB/HER receptors form regulatory complexes in which components can enter the cytoplasm and promote downstream molecular pathways, including oncogenic signaling axis of RAS-RAF-MEK-ERK and AKT-PI3K-mTOR (Shaban et al., 2023).

Abnormal expression and dysregulated intracellular signaling through the HER family members play a pivotal role in the carcinogenesis of many human malignancies, including head and neck (Kalyankrishna and Grandis, 2006), lung (Bethune et al., 2010), breast (Al-Kuraya et al., 2004), pancreatic (Oliveira-Cunha et al., 2011), and colon cancer (Pabla et al., 2015). Specifically, HER2 overexpression serves as a prognostic and predictive biomarker in many types of cancer, including breast cancer (BC). Amplification or overexpression of HER2 occurs in approximately 15%–30% of BC cases and 10%–30% of gastric/gastroesophageal cancers (Iqbal and Iqbal, 2014).

Targeted anti-HER2 drugs have been used in HER2+ breast cancer patients for several decades. The humanized monoclonal antibodies trastuzumab and pertuzumab, which target extracellular domains of HER2, have been approved as the standard therapeutic of the HER2+ BC (Ding et al., 2020). Synthetic small molecule tyrosine kinase inhibitors (TKIs) have also been developed for the treatment of HER2+ BC: for example, lapatinib, a reversible first generation TKI, which prevents phosphorylation and activation of both HER2 and EGFR (Gril et al., 2008; Kim et al., 2009; Yuan et al., 2022), has been proven to be clinically effective against the HER2+ BC (Bilancia et al., 2007). A second generation TKI, neratinib, which irreversibly binds to the kinase domain of HER1, HER2, and HER4 (Wissner and Mansour, 2008), has been approved for use in early-stage HER2+ BC patients who have already undergone a one-year course of trastuzumab treatment (Singh et al., 2018).

However, not all the patients with tumors expressing high levels of HER2 respond to the HER2-targeted therapy (Figueroa-Magalhaes et al., 2014; Piccart-Gebhart et al., 2005; Slamon et al., 2011), and frequently tumors become resistant to such treatment and develop progressive disease after an average of 12 months (Vu and Claret, 2012; Wang et al., 2022) due to either initially present or acquired factors (Amir et al., 2010). HER2-therapy resistance is associated with downstream signal activation by compensatory pathways, mutations in the HER2-TK domain, tumor stem cell self-renewal, host immune regulation, and epigenetic effects (D’Amato et al., 2015; Xing et al., 2023). For instance, only 39% of the patients with HER2+ inflammatory breast cancer responded to lapatinib (Kaufman et al., 2009). Moreover, it has been found that tumor cells resistant to one HER2-targeted drug are also cross-resistant to other drugs: cells resistant to lapatinib and trastuzumab are also resistant to neratinib (Breslin et al., 2017).

Efforts to discover and validate effective and clinically relevant factors related to drug resistance are ongoing to further refine treatment efficacy. Intracellular factors are characteristic of the tumor itself and defined by mutations or abnormal gene expression. Some BC patients with HER2 mutations (L755S, V842I, K753I, or D769Y) do not seem to benefit from trastuzumab (Gaibar et al., 2020). Several groups of putative response factors are discussed in the literature, including tumor mRNA expression biomarkers (Bao et al., 2020), and transcriptome-based deduced activities of intracellular molecular pathways in the tumor tissues (Havaleshko et al., 2009; Triulzi et al., 2015; von der Heyde et al., 2015).

Candidate biomarkers for the efficacy of HER2 receptor therapy were also identified by proteomic analysis of cell lines with different HER2 gene amplifications (Tang et al., 2013). In another study the heterogeneity of breast cancer by extracellular matrix, lipid metabolism, and immune-response features was characterized by proteomic analysis of formalin fixed paraffin embedded (FFPE) tissue specimens with extended clinical outcomes (Asleh et al., 2022). Proteomic and phosphoproteomic profiling of pre-treatment biopsies of patients with early-stage HER2+ BC were performed to identify multiple cellular mechanisms that precondition tumors to resist therapy by trastuzumab, pertuzumab and chemotherapy. Among them are unfolded protein response (UPR) induced cellular dormancy, a metabolic switch toward oxidative phosphorylation (OXPHOS), and reduced numbers of tumor-infiltrating leukocytes (TILs) (Debets et al., 2023). Proteomic analysis of the BT474 cell line following treatment with different HER2 inhibitors highlighted several proteins that are closely associated with early HER2-inhibitor response. In particular, the proteins like trifunctional enzyme subunit alpha, mitochondrial; heterogeneous nuclear ribonucleoprotein R; LAP2α and HSC70 that were altered in abundance in three or more comparisons and may be strongly involved in an early treatment response to HER2-inhibition were identified (Di Luca et al., 2015).

Extracellular factors associated with the drug resistance relate to cellular communication or factors present in the patient body. To identify such factors, comparison of the proteomic profiles of the blood serum samples of responders and non-responders was performed (Taguchi et al., 2007; Yang et al., 2020). Certain indications suggest that HER and its ligands (i.e., epidermal growth factor (EGF), amphiregulin, HB-EGF, TGF-alpha) could be used as serological biomarkers for prognosis and prediction of response to HER-targeted treatments. In the cell culture studies, strong rescuing effects of HER ligands (EGF, neuregulin (NRG)) on cells treated with HER-targeted drugs, including lapatinib, were demonstrated (Claus et al., 2018; Kamashev et al., 2021; Wang et al., 2016; Wilson et al., 2012; Kamashev et al., 2022).

Thus, tumor response could be affected by a variety of extracellular factors present in human peripheral blood. Nonetheless, crosstalk between the human serum and targeted drugs has not yet been sufficiently investigated. The molecular markers and associated phenotypic traits observed in breast cancer cell lines including SK-BR-3 are also frequently discriminative features in tumors (Dai et al., 2017). Recently we found that human blood serum dramatically abolishes the lapatinib-mediated growth inhibition of the human breast squamous carcinoma SK-BR-3 cell line. This antagonism was associated with cancelation of the drug induced G1/S cell cycle transition arrest. RNA sequencing demonstrated that in the presence of human serum or EGF, lapatinib was unable to alter the Toll-Like Receptor signaling pathway and alter the expression of genes linked to Focal adhesion (Shaban et al., 2024). Here we performed proteomic profiling of the SK-BR-3 cells treated with lapatinib in the presence of human sera or EGF. We found that in addition to the abovementioned processes lapatinib treatment causes activation of cell respiration, which was confirmed experimentally in the resazurin assay.

## 2 Materials and methods

### 2.1 Materials

Lapatinib (Sigma-Aldrich, United States) was diluted to a stock concentration of 10 mM in DMSO and stored at −20°C. Lyophilized recombinant human EGF (rhEGF) was purchased from SCI-Store (Russia) and stored at −20°C. For comparison with human serum the following animal sera were used: goat serum (Capricorn Scientific, Germany), horse serum (Capricorn Scientific, Germany), fetal bovine serum (FBS) (Biosera, France), and bovine serum (PanEco, Russia).

### 2.2 Human blood serum samples

Peripheral blood samples from 10 unrelated healthy donors aged 23–64 years were collected into two 8 mL Vacuette serum tubes (Greiner Bio-One, Austria). Serum was separated within 3–12 h after blood collection by centrifugation at 2,500 rpm for 15 min. Serum samples were aliquoted and stored at −75°C before use. Informed written consent for participation in the study and transmission of results in the form of a scientific report was obtained from all donors. The study was conducted in accordance with the Helsinki Declaration; the consent procedure and study design were approved by the ethics committee of the Vitamed Medical Center, Moscow; approval date: 6 October 2021.

To investigate the influence of serum on the activity of lapatinib, two random individual samples (DS12, DS19) and a pooled sample from all 10 donors (DSP) were used.

### 2.3 Determining the concentrations of EGF in human serum samples

The level of EGF in serum was determined using the Human EGF DuoSet ELISA kit (R&D Systems, United States) by the enzyme-linked immunosorbent assay (ELISA) method in accordance with the manufacturer’s recommendations, performed in at least three independent replicates. Serum samples were diluted to 4% using the Reagent Diluent. The optical density at wavelengths of 450 and 550 nm was measured using a microplate reader Varioskan Flash (Thermo Scientific, United States).

### 2.4 Cell culture

SK-BR-3 human breast cancer cell line (ATCC HTB-30) was obtained from the collection of the Institute of Cytology, St. Petersburg, Russia. SK-BR-3 cells were cultured at 37°C in a humid atmosphere with 5% CO2 in RPMI-1640 medium (Paneco, Russia) supplemented with 10% fetal bovine serum (Biosera, France), 2 mM L-glutamine, 4.5 g/L glucose, and 1% penicillin-streptomycin mixture (Paneco, Russia).

### 2.5 Cell growth rate measurement

Cells were seeded into 24-well culture plates at approximately 5,500 cells per well in 0.5 mL growth medium. The plates were incubated for 24 h before treatment with lapatinib, with or without the addition of rhEGF or human blood serum. On the day of drug addition, a baseline cell count was determined. Six days after drug treatment, the medium was removed, cells were washed and trypsinized for 10 min. Following resuspension, cell numbers were determined using a Neubauer Improved cell counting chamber. Cell growth (%) was calculated as the increase in treated cell number normalized to the increase in cell number in the control wells. All experiments were performed in independent triplicates (on different days from different batches of cells). In the preliminary studies we counted the cells each day during the time-course treatment. When cells are treated for more than 6 days, the control well reaches confluence. When cells are treated for less than 6 days, the ratio between the cell count and a baseline cell count (on the day of drug addition) decreases. This reduction occurs because lapatinib treatment causes cell growth arrest without leading to cell detachment and death.

### 2.6 Resazurin-based cellular respiration assay

Cells were seeded into 96-well culture plates at approximately 4,100 cells per well in 0.15 mL of the growth medium. The plates were incubated for 24 h before treatment with lapatinib, with or without the addition of rhEGF or human blood serum. Two days after drug addition, the medium was removed, and a resazurin solution in PBS (0.1 mL per well) was added to the cells. The plates were then incubated at 37°C in the dark for 3 h to allow the accumulation of the resazurin reduction product – resorufin. The intensity of resorufin fluorescence was measured at an emission wavelength of 590 nm with an excitation wavelength of 550 nm using a microplate reader Varioskan Flash (Thermo Scientific, United States).

### 2.7 Cell samples preparation for proteomic study

14 mL of SK-BR-3 cell suspension (36,400 cells/mL) was seeded to 75 cm^2^ culture flasks. Flasks were incubated for 16 h prior to the addition of lapatinib with or without the addition of rhEGF or human blood serum. Immediately before adding lapatinib, it was diluted in the growth medium without FBS to a final concentration of 150 nM, while rhEGF was diluted to a final concentration of 2 ng/mL. Human blood serum was added to corresponding flasks at a volume of 740 µL (to a final concentration of 5%). After the addition of the substances, the cells were incubated for 48 h, after which the medium was removed, cells were detached from the substrate with trypsin, washed with PBS, and pelleted by centrifugation. Cell pellets were stored at −70°C before the proteomic analysis.

For the control measurement of growth rate, aliquots of 0.5 mL from the same cell suspension were added to each well of the 24-well plates and growth rate were deter-mined as described in the Section 2.5.

### 2.8 Cell lysis, protein extraction, protein concentration measurement

To cell pellets, 50 µL of a 2% solution of sodium deoxycholate was added, followed by sonication using an ultrasonic disintegrator with a Bandelin Sonopuls probe (BAN-DELIN electronic GmbH KG, Germany) at 70% power for 1 min on ice. Lysates were centrifuged for 5 min at 10,000 g and 15°C. The supernatants were transferred to new tubes. The protein concentration was determined by Pierce BCA assay kit (Thermo Scientific, United States) following the manufacturer’s recommendations (Walker, 1994).

### 2.9 Protein hydrolysis using the S-Trap protocol

For protein hydrolysis with trypsin using the S-Trap method, 100 µg of each sample was selected. To reduce and alkylate disulfide bonds, samples were incubated in the presence of 4 mM tris(2-carboxyethyl)phosphine (TCEP) and 6.2 mM chloroacetamide (CAA) at 80°C for 30 min. Then, the samples were cooled to room temperature, and 12% H3PO4 was added at a ratio of 1:10 by volume and were pipetted. The resulting solutions were combined with 6 parts of S-Trap protein binding buffer (90% methanol in 100 mM triethylammonium bicarbonate buffer (TEAB), pH 7.5), thoroughly mixed, and transferred to the S-Trap filters. After that, tubes with S-Trap filters were centrifuged for 4 min at 4,000 g (repeated until the samples were completely applied). The filters were washed four times with S-Trap protein binding buffer, applying 150 µL of buffer each time and centrifuging for 4 min at 4,000 g. Then, the filters were transferred to clean tubes, and 40 µL of hydrolysis buffer (50 mM TEAB, pH 8.5, containing trypsin at a ratio of enzyme to protein = 1:25) was added. The samples were incubated for 1.5 h at 47°C. After that, 40 µL of 50 mM TEAB with 0.2% formic acid was added to the S-Trap filters, and they were centrifuged again for 4 min at 4,000 g. The filters were transferred to new tubes. For elution of tryptic peptides, 35 µL of 50% acetonitrile containing 0.2% formic acid was applied to the filters, and centrifugation step was repeated. The eluates were transferred to glass vials and dried in a rotary evaporator at 45°C. After complete drying, the samples were reconstituted in HPLC-grade water for total peptide measurement using the Pierce Quantitative Colorimetric Peptide Assay kit (Thermo Scientific, United States). Peptides were dried again and dissolved in 0.1% formic acid to a final concentration of 3 μg/μL.

### 2.10 LC-MS/MS data aquisition

The obtained peptides were analyzed using the UltiMate 3000 RSLCnano chroma-tographic system (Thermo Scientific, United States) and Q-Exactive HFX mass spectrometer (Thermo Scientific, United States). One microliter of peptide mixture was loaded onto an Acclaim µ-Precolumn (0.5 mm × 3 mm, particle size 5 μm, Thermo Scientific, United States) at a flow rate of 10 μL/min for 4 min in isocratic mode using 2% acetonitrile, 0.1% formic acid in deionized water as the mobile phase. Subsequently, peptides were separated on a analytical PeakyEfficiency nano-LC column (FE 100 μm × 50 cm, particle size 1.9 µm, Molecta, Russia) in gradient elution mode at a flow rate of 0.3 μL/min. Two mobile phases (MP) were used: MP A (0.1% formic acid) and MP B (80% acetonitrile, 0.1% formic acid). The column was washed with 2% MP B for 10 min, followed by a linear increase to 35% MP B over 68 min, then to 99% MP B over 2 min, after which the concentration of MP B was linearly reduced to the initial 2% over 3 min. The total duration of the analysis was 90 min. Peptides detection was performed on a Q-Exactive HFX mass spectrometer in positive ionization mode using the NESI source (Thermo Scientific, United States) with emitter voltage 2.1 kV and capillary temperature 240°C. Full scan was performed in the mass range from 300 m/z to 1,500 m/z, with a resolution of 1,20,000. For tandem scanning, the resolution was set to 15,000 in the mass range from 100 m/z to the precursor mass, but not exceeding 2,000 m/z. Precursor ions were isolated in the window of ± 1 Da. The maximum number of ions allowed for isolation in MS2 mode was set to no more than 40, with a precursor selection cut-off of 50,000 units, and the normalized collision energy (NCE) was set to 29. Tandem scanning considered only ions with charges from 2+ to 6+. The maximum accumulation time for precursor ions was 50 ms, and for fragment ions - 110 ms. The AGC value for precursor and fragment ions was set to 1 × 106 and 2 × 105, respectively. All measured precursor ions were dynamically excluded from tandem MS/MS analysis for 90 s.

### 2.11 Protein identification

Protein identification was performed using MaxQuant v. 2.0.3.0 software with the Andromeda search algorithm (Tyanova et al., 2016a). The UniProt human proteome database (UP000005640) was utilized for protein identification. The following search parameters were set: trypsin as the cleaving enzyme, ±4.5 ppm mass accuracy for monoisotopic peptide detection, ±20 ppm mass accuracy for MS/MS spectra, and allowance for up to two missed trypsin cleavage sites. Methionine oxidation, N-terminal acetylation, and cysteine carbamidomethylation were considered as potential and mandatory peptide modifications, respectively. The “match between runs” option was applied during identification with default settings. A False Discovery Rate (FDR) of less than 1.0 was used for validation of spectrum-peptide matches (PSM), peptide identifications, and protein identifications. Proteins were considered confidently identified if at least two peptides were detected. Label-free quantification (LFQ) was employed for protein quantification with Perseus (Tyanova et al., 2016b).

### 2.12 Bioinformatic analysis of proteomic data and statistical analysis

Prior to analysis, data were filtered to remove potential contaminating proteins, false-positive identifications, and to retain proteins identified by at least two peptides present in three technical replicates of at least one sample. Statistical significance was defined as a q-value <0.05 and |log2(fold change)| >2. Differential expression analysis was performed using non-parametric Mann Whitney U-test on log2-transformed LFQ intensities; *p*-values were adjusted according to Benjamini and Hochberg false discovery rate correction procedure with the statistical threshold of 0.05.

Gene Ontology (GO) enrichment analysis was conducted using R packages clusterProfile (v.4.2.1) and org.Hs.e.g.,.db (v.3.8.2). Pathways and GO terms were filtered using q-values < 0.05 as the cutoff threshold. GO terms were visualized using the R package enrichplot (http://bioconductor.org/packages/ release/bioc/html/enrichplot.html). Pathway activation levels (PALs) were calculated and visualized using the OncoboxPD toolkit (Zolotovskaia et al., 2022). Molecular functions of 3,044 pathway components were algorithmically annotated according to (Sorokin et al., 2021; Sorokin et al., 2020) and pathways with more than 10 participants were selected for further analysis (1,529 pathways). For PAL calculations, each sample expression profile was normalized on mean geometrical levels of protein expression for all samples in the dataset under analysis. Significance of intersections was assessed as per (Raevskiy et al., 2022). Signal pathways were visualized using the OncoboxPD toolkit (Borisov et al., 2020).

Statistical analysis of growth rate change was performed using GraphPad PRISM 6.0 (GraphPad Software Inc.), with *p*-values < 0.05 considered statistically significant. Data are presented as mean ± SD from at least three experiments conducted on different days.

## 3 Results

In this study, we investigated the influence of human blood serum on the efficacy of the EGFR-targeted drug lapatinib, a dual reversible inhibitor of tyrosine kinase activity of ERBB1 (EGFR) and ERBB2 (HER2). To achieve this, we measured the growth rate of HER2+ SK-BR-3 cells in media supplemented with lapatinib and various serum types, including human blood serum, animal serum, or EGF. To explore the molecular mechanisms underlying this effect, we conducted proteomic profiling of SK-BR-3 cells. Differentially expressed proteins were identified, and the activation of molecular pathways was analyzed. Resazurin test was performed to measure endogenous NAD(P)H concentrations.

### 3.1 Human serum abrogates the effect of lapatinib on SK-BR-3 cell growth

The breast cancer cell line SK-BR-3 has increased expression of HER2 (with approximately 1.5 × 10^6^ HER2 protein molecules per cell, compared to ∼4.0 × 10^4^ and ∼2 × 10^4^ in HER2-negative cell lines MDA-MB-231 and A431, respectively (Sato et al., 2013; Shaban et al., 2024). Thus, SK-BR-3 cells are highly sensitive to EGFR-targeted drugs and are widely used in studies of their activity. Categorization, molecular information and culture conditions of SK-BR-3 (ER-, PR-, HER2+) and other 83 BC cell lines, and the clinical features of tumors where they derive are described in (Dai et al., 2017).

The IC50 of lapatinib in our tests (in the presence of 10% FBS) is approximately 45 nM. For comparison, the IC50 values of lapatinib for MDA-MB-231 and A431 are two to three orders of magnitude higher: 27 μM and 10 μM, respectively (Shaban et al., 2024).

In this study, to investigate the interaction of human blood serum with the targeted drug lapatinib, we initially examined the effect on proliferation of SK-BR-3 cells. First, we tested whether human serum itself affected SK-BR-3 growth. Because the efficacy of therapeutic drugs can be influenced by various molecular factors present in the patient’s body, we tested both sera from individual donors (DS12, DS19) and a pooled serum sample from 10 healthy donors (DSP). We additionally tested various animal sera (fetal bovine, bovine, horse, pig, and goat) to identify possible species-specific effects. For all tested samples, the initial growth medium contained 5% of heat-inactivated (30 min at 56°C) fetal bovine serum (hiFBS). The test sera were added to this medium to the concentration of 5%.

**FIGURE 1:**
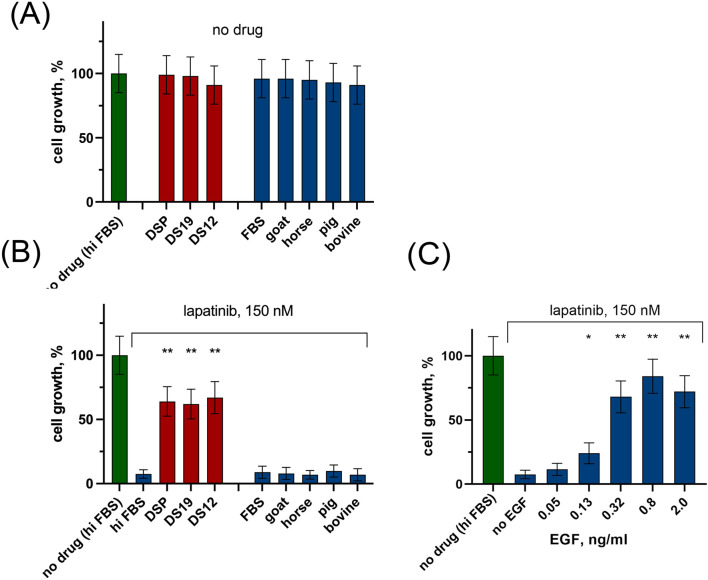
The influence of human serum, animal sera, and rhEGF on growth rate of SK-BR-3 cells in the presence and in the absence of lapatinib. Human serum (individual DS12 and DS19, as well as pooled DSP) and animal sera (bovine, goat, horse, pig) were added at a concentration of 5% to the growth medium containing 5% heat inactivated FBS (hiFBS). Cell number was determined after 6 days of incubation. Growth rate was normalized to control wells with 10% hiFBS (no drug (hiFBS)). **(A)** without lapatinib; **(B)** in the presence lapatinib. **(C)** The effect of rhEGF on the growth of SK-BR-3 cells in the presence of 150 nM lapatinib. The columns represent the average cell growth rate for each sample calculated for three replicates and normalized to the no drug control. Asterisks show the statistical significance of the overlaps for the lapatinib – treated cells: ^*^ *p* < 0.05; ^**^ *p* < 0.01; ^***^ *p* < 0.001.

Cell growth was assessed by quantifying the increase in cell numbers after 6 days of incubation with drugs compared to control conditions (full growth medium with 10% hiFBS, depicted by the green column in Figure 1). As shown in Figure 1A, none of the tested sera exhibited a statistically significant effect on cell growth. This finding aligns with the results of our previous study, where 14 blood serum samples from healthy donors yielded similar outcomes (Shaban et al., 2024).

Second, we determined the effect of lapatinib on cell growth rate in the presence of 5% of human or animal serum. Both individual and pooled samples of human serum significantly prevented lapatinib-induced inhibition of cell growth (Figure 1B). The average growth rate was 7.5% ± 3.4% for 150 nM lapatinib alone, and ∼65% when lapatinib was added together with human serum (67.0% ± 12.5% for DS12, 62.0% ± 11.6% for DS19, and 64.0% ± 11.5% for DSP). In contrast, the addition of an equal amount of animal blood serum to the growth medium did not significantly affect the action of lapatinib.

### 3.2 Antagonistic effect of EGF on lapatinib inhibition of HER2-positive breast cancer cell proliferation

Human blood serum contains ligands of HER2 and EGFR, including EGF. It was previously demonstrated that cancer cells’ sensitivity to tyrosine kinase inhibitors can be diminished by exposing them to RTK ligands. In particular, EGF and another ERBB ligand, Heregulin1 (HRG1), could protect cancer cells from action of lapatinib (Claus et al., 2018; Kamashev et al., 2021; Wang et al., 2016; Wilson et al., 2012; Canadas et al., 2014; Sato et al., 2013). We also recently demonstrated that adding recombinant human EGF (rhEGF) to the growth medium at concentrations ranging from 0.82 to 20 ng/mL prevented the inhibition of SK-BR-3 growth by lapatinib (Shaban et al., 2024). However, the concentration of EGF in our previous work and publications by other authors significantly exceeded the physiological values of EGF concentration in human blood, which ranges from 0.3 to 1.7 ng/mL (Kjaer et al., 2019; Lemos-Gonzalez et al., 2007).

To determine whether the presence of endogenous EGF in human blood serum samples explains the serum effect on lapatinib action, we 1) measured the concentration of EGF in tested serum samples, and 2) measured the cell growth rate in the presence of corresponding concentrations of rhEGF.

The concentration of EGF in the sera used in the experiments was measured with the Human EGF DuoSet ELISA kit (R&D Systems, United States), yielding 0.677 ng/mL for sample DS12, 0.881 ng/mL for sample DS19, and 0.755 ng/mL for the DSP serum sample pooled from 10 individual sera. Therefore, considering the 20-fold final dilution of sera in the medium, we selected a range for testing the effect of rhEGF equal to 0.05–2 ng/mL.

Figure 1C illustrates that a concentration of 0.05 ng/mL of rhEGF did not produce a statistically significant impact on the efficacy of lapatinib. Hence, the presence of EGF in the analyzed human serum samples most likely does not account for the observed protective effect on tumor cells.

### 3.3 The impact of lapatinib on the proteomic profile of SK-BR-3 cells in the presence of human serum or EGF

To assess the molecular mechanisms underlying the serum-mediated and/or EGF-mediated prevention of lapatinib-induced growth arrest in SK-BR-3 cells, we conducted the proteomic profiling. Twelve cell samples were analyzed after 48 h incubation: 1) in standard medium (control); 2) with 150 nM lapatinib; 3) with 2 ng/mL rhEGF; 4) with 5% human blood serum; 5) with 150 nM lapatinib + 2 ng/mL rhEGF; 6) with 150 nM lapatinib + 5% human blood serum. The cell growth conditions were identical to those recently used for transcriptomic profiling and cell cycle analysis (Shaban et al., 2024).

Samples were lysed, proteins were extracted according to S-Trap protocol, and the total protein concentration was measured. Subsequently, 100 μg of samples were subjected to trypsin hydrolysis. The resulting tryptic peptides were analyzed using LC-MS/MS on the UltiMate 3000 RSLCnano chromatographic system (Thermo Scientific, United States) and Q-Exactive HFX mass spectrometer (Thermo Scientific, United States). The analysis was carried out in three technical replicates for each sample.

As a result of the proteomic analysis of 12 samples, excluding contaminants (n = 30), a total of 3,947 proteins, meeting the criteria for reliable identification (at least 2 identified peptides per protein), were determined. The average number of identified proteins was 3,847 ± 20 (Table 1).

**TABLE 1:** The number of proteins identified in the proteomic analysis for SK-BR-3 cells after 48 h incubation with lapatinib, rhEGF and human serum.

Cell treatment	Number of identified proteins
Control, no drug	3,839
5% human serum	3,865
2 ng/mL rhEGF	3,825
150 nM lapatinib	3,878
150 nM lapatinib + 5% human serum	3,864
150 nM lapatinib + 2 ng/mL rhEGF	3,836

We further identified differentially expressed proteins (DEPs) for the above conditions compared to the control samples. Obtained fold change (FC) values of DEPs (with criteria of FDR-adjusted *p*-value < 0.05; |log2(fold change)| >2) are provided in Supplementary Table S1. The mass spectrometry proteomics data have been deposited to the ProteomeXchange Consortium via the PRIDE repository with the dataset identifier 1-20240724-81057.

**FIGURE 2:**
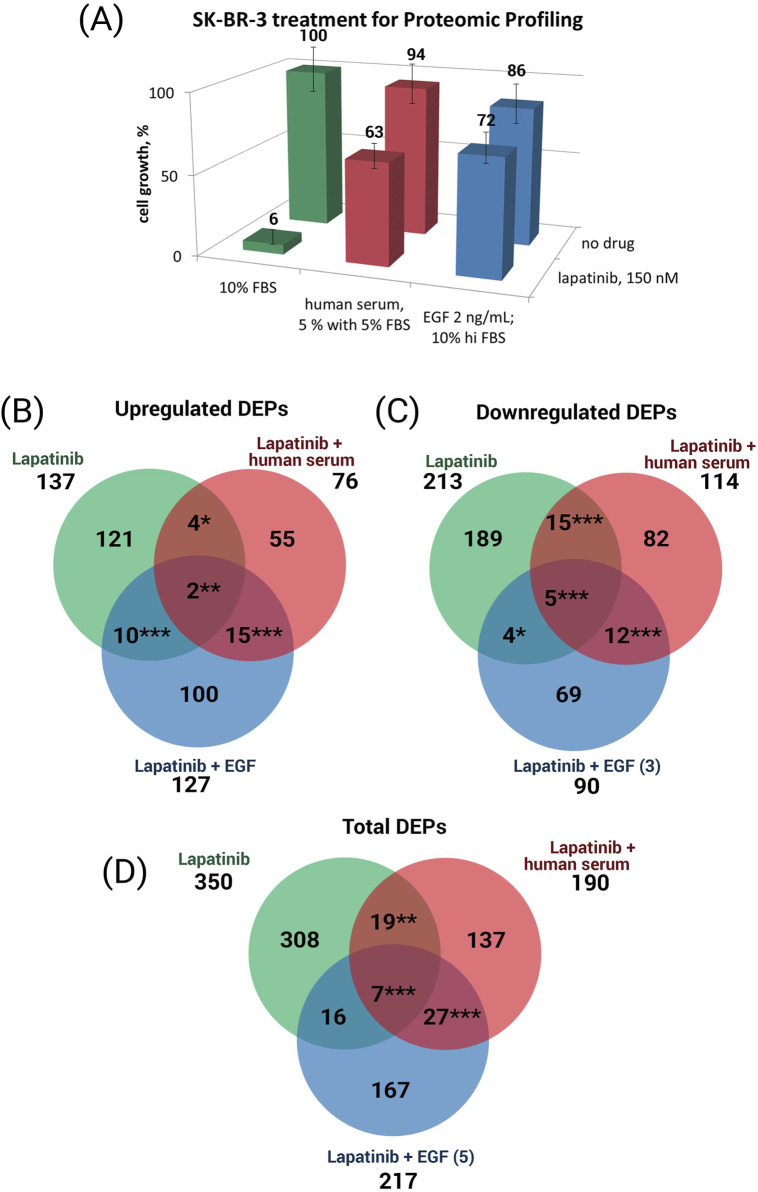
**(A)** SK-BR-3 cell growth under the cell treatment conditions used to collect samples for proteomic analysis. Venn diagrams showing the overlap in the upregulated **(B)** and downregulated **(C)** proteins, and both upregulated plus downregulated proteins **(D)**, in the presence of lapatinib only or lapatinib plus human serum, or lapatinib plus EGF. Asterisks show the statistical significance of the overlaps: ^*^ *p* < 0.05; ^**^ *p* < 0.01; ^***^ *p* < 0.001.

We found that a 48 h treatment of SK-BR-3 cells with 150 nM lapatinib reduced the growth rate of SK-BR-3 to 6% compared to the no-drug conditions and caused differential expression of 350 proteins, of which 137 were up-regulated and 213 were down-regulated (Figure 2). When lapatinib was combined with EGF, these changes in protein expression were diminished: out of initial 350 DEPs the expression of only 23 proteins remained altered (Figure 2D). The addition of EGF to the lapatinib resulted in cell growth recovery to 72% of the control level without the drug. When human blood serum was added along with lapatinib, these changes in protein expression were also greatly reduced: out of initial 350 DEPs the expression of only 26 remained altered (Figure 2D). The addition of human serum along with lapatinib resulted in cell growth recovery to 63% of the control level without the drug.

We identified a set of 308 lapatinib-specific DEPs whose expression is affected by the lapatinib only treatment (cell growth ceased) and not affected when the cell growth media contains lapatinib along with EGF or human serum (cell growth is restored); we define these 308 proteins as the core lapatinib proteins (Figure 2D, DEPs in green color). The expression of these proteins is most probably associated with the cell growth rate inhibition by lapatinib treatment.

Remarkably, among the 121 upregulated *lapatinib core* proteins, 50 (41%) are associated with mitochondrial function, tricarboxylic acid cycle (TCA), and electron transport chain (Supplementary Table S1). Proteins which belong to GO terms “mitochondrial inner membrane” and “aerobic respiration” are listed in the Table 2.

**TABLE 2:** *Lapatinib core* induced proteins associated with mitochondrial function^a^.

Symbol	Protein name	Function^b^	log2FC	GO term^c^
ACADVL	Acyl-CoA dehydrogenase very long chain	M	2, 1	24
AIFM1	Apoptosis inducing factor mitochondria associated 1	M	2, 2	8
CHCHD1	Coiled-coil-helix-coiled-coil-helix domain containing 1	M	2, 2	12
CYC1	Cytochrome C1	M; R; E	2, 2	15
GOT2	Glutamic-oxaloacetic transaminase 2	M	2, 4	25
HADHA	Hydroxyacyl-CoA dehydrogenase subunit alpha	M	2, 3	19
HADHB	Hydroxyacyl-CoA dehydrogenase subunit beta	M	2, 2	21
IMMT	Inner membrane mitochondrial protein	M	2, 2	9
LETM1	Leucine zipper and EF-hand containing transmembrane protein 1	M	2, 1	3
NDUFA10	ADH:Ubiquinone oxidoreductase subunit A10	M; R; E	2, 1	41
NDUFB8	NADH:Ubiquinone oxidoreductase subunit B8	M; E	2, 0	41
NDUFB11	NADH:Ubiquinone oxidoreductase subunit B11	M	2, 2	36
OPA1	OPA1 mitochondrial dynamin like GTPase	M	2, 2	15
PMPCA	Peptidase, mitochondrial processing subunit alpha	M	2, 1	4
SDHA	Succinate dehydrogenase complex flavoprotein subunit A	M; R; E	2, 1	41
SLC25A13	Calcium-binding mitochondrial carrier protein aralar2	M; E	2, 1	31
CAT	Catalase	R	2, 1	61
FH	Fumarate hydratase, mitochondrial	R	2, 0	7
MDH2	Malate dehydrogenase, mitochondrial	R	2, 1	7
OGDH	2-oxoglutarate dehydrogenase, mitochondrial	R	2, 1	27
PDHA1	Pyruvate dehydrogenase E1 component subunit alpha, mitochondrial	R	2, 4	31
PDHB	Pyruvate dehydrogenase E1 component subunit beta, mitochondrial	R	2, 4	31
SUCLA2	Succinyl-CoA ligase [ADP-forming] subunit beta, mitochondrial	R	2, 0	22
SUCLG2	Succinyl-CoA ligase [GDP-forming] subunit beta, mitochondrial	R	2, 2	21

^a^ 24 of 50 lapatinib core activated proteins associated with mitochondrial function (only proteins which belong to GO terms “mitochondrial inner membrane” and “aerobic respiration” are listed). For each protein the FDR-adjusted *p*-value < 0.002.

^b^ M, associated with mitochondrial inner membrane; R, cellular respiration; E, electron transport chain.

^c^ Number of lapatinib altered GO terms containing the protein detected.

The following proteins are among the major lapatinib-induced proteins linked to the mitochondrial oxidation process within the cell.

Acyl-Coenzyme A dehydrogenases (ACADM - medium-chain specific and ACADVL - very long-chain) catalyze the initial step of the mitochondrial fatty acid beta-oxidation pathway (Ma A. P. Y. et al., 2021).

Acetyl-CoA acetyltransferase 1 (ACAT1) is a mitochondrial enzyme that catalyzes the reversible formation of acetoacetyl-CoA from two acetyl-CoA molecules. ACAT1 overexpression suppresses proliferation and migration of human renal carcinoma cells *in vitro*. Patients with reduced ACAT1 expression have significantly lower overall survival and disease-free survival (Chen et al., 2019).

Aldehyde dehydrogenases (ALDH4A1 and ALDH6A1) overexpression has been shown to be associated with poorer overall patient survival, particularly in breast, head and neck, cervical and ovarian cancers (Xia et al., 2023).

2,4-Dienoyl-CoA reductase (DECR1) is an enzyme involved in the beta-oxidation and metabolism of polyunsaturated fatty enoyl-CoA esters (Nassar et al., 2020). DECR1 expression is significantly reduced in numerous breast tumor models and in primary breast cancer. Moreover, ectopic expression of DECR1 in ErbB2/Neu-induced breast tumor cells is sufficient to reduce ErbB2/Neu expression levels and impair breast tumor growth. DECR1 is sufficient to restrict breast cancer cell proliferation through its ability to limit the extent of oncogene expression and reduce the stable level of *de novo* fatty acid synthesis (Ursini-Siegel et al., 2007). Thus, the upregulation of DECR1 in cells upon lapatinib impact may contribute to cell growth arrest.

Malate dehydrogenase (MDH2) is an enzyme that catalyzes the reversible oxidation of malate to oxaloacetate in the TCA using the NAD/NADH cofactor system and cellular respiration. Malate dehydrogenase expression is upregulated in lung cancer samples, and knockdown of this gene in tumor cells suppressed their proliferation (Ma Y. C. et al., 2021). Inhibition of MDH2 reduces NADH levels and cellular ATP production in colorectal cancer cells, which in turn inactivates ACC and mTOR signaling pathways (Ban et al., 2016).

Fumarase hydratase (FH) is an enzyme that catalyzes the reversible hydration and dehydration of fumarate to malate in the TCA cycle. Low FH expression has been shown to activate oncogenic signaling cascades in cancer cells and promote metastasis, and patients with low FH expression have a shorter overall survival (Vadhan et al., 2023; Schmidt et al., 2020). In contrast, increased expression of this protein reduces lung cancer cell migration (Vadhan et al., 2023). When FH was overexpressed in endometrial cancer cells, the proliferative, migratory and invasive capacity of these cells was reduced, accompanied by partial inactivation of EGFR (Wang et al., 2023).

Increased expression of some mitochondrial ribosomal proteins (MRPL42, MRPS11, MRPS15 and MRPS18A) was detected in lapatinib-treated cells. Dysregulation of mitochondrial ribosomal genes is common in various neurodegenerative and autoimmune diseases as well as in tumors (Sorensen et al., 2017; Sotgia et al., 2012; Najafzadeh et al., 2021). Mitochondrial ribosomal protein L42 (MRPL42) is a component of both subunits of mitochondrial ribosomes. Its increased expression is seen in various cancers such as lung cancer and glioma. Reduced expression of this protein leads to inhibition of metastasis and proliferation of tumor cells, inducing cell cycle arrest and apoptosis (Hao et al., 2018; Jiang et al., 2021).

The OGDH protein is a subunit of the mitochondrial 2-oxoglutarate dehydrogenase (OGDHC) complex that catalyzes the conversion of 2-oxoglutarate to succinyl-CoA and CO2 during the tricarboxylic acid cycle. This protein is a limiting protein in the tricarboxylic acid cycle. Increased expression of OGDH also activates the Wnt/β-catenin signaling pathway and promotes oncogenesis (Lu et al., 2019; Meng et al., 2021).

It is known that inhibition of EGFR and HER2 activation significantly affects redox regulation mechanisms in cancer cells (Chang et al., 2011; Rodriguez-Hernandez et al., 2020) and may lead to mitochondrial dysfunction. This was hypothesized to be the cause of side effects of HER targeting therapies. However, by proteomic profiling we found no changes in the expression of key redox regulators (including thioredoxin 1 (Trx1) and glutaredoxin 1 (Grx1), peroxiredoxin 6 (Prdx6), and thioredoxin reductase 1 (TrxR1), peroxiredoxin 2 (Prdx2)). We also found that the expression of cytochrome C1 (CYC1) protein, which is involved in the mitochondrial respiratory chain, was induced by lapatinib. Catalase (CAT) is a key antioxidant enzyme in the biogenic defense against oxidative stress that converts reactive oxygen species into water and oxygen, thus attenuating their toxic effects, is also induced by lapatinib.

Among the 189 lapatinib core proteins whose expression is downregulated by lapatinib, 80 are structural ribosomal proteins, translation-related proteins, chaperones and heat shock proteins (Supplementary Table S1).

Recently we reported results of the transcriptome profiling of SK-BR-3 cells treated with lapatinib, EGF, and human blood serum (Shaban et al., 2024). We performed a correlation analysis of transcriptome profiling and proteome profiling data for each treatment condition (Table 3). The calculated Spearman correlation coefficient was in the range of 0.45–0.49 with *p*-values less than 10^−191^. Overall, this indicates a well match of the gene and protein expression data and is in line with the previous comparisons (correlation coefficients between transcriptome and proteome profiles in the range of 0.3–0.5 (Jiang et al., 2020; Ponomarenko et al., 2023)).

**TABLE 3:** SK-BR-3 cell line proteome/transcriptome correlation analysis.

Cell treatment	R_spr^a^	*p*-value
Control, no drug	0.487	5.66e-234
5% human blood serum	0.493	3.72e-239
2 ng/mL rhEGF	0.491	1.52e-234
150 nM lapatinib	0.448	4.35e-191
150 nM lapatinib + 5% human blood serum	0.479	1.56e-227
150 nM lapatinib + 2 ng/mL rhEGF	0.456	7.53e-199

^a^ R_spr, Spearman correlation coefficient.

Most studies consider the mRNA level of gene expression as transcriptomic methods are more standardized, representative, and less laborious than proteomic methods (Buzdin et al., 2021). However, as we demonstrate here, it was only through proteomic analysis that we were able to discern the activation of mitochondrial respiration pathway following lapatinib treatment, a finding that was not evident at the transcriptomic level. Thus, it is crucial to consider differential expression on both proteomic and transcriptomic levels when assessing the impact of drug influence (Jiang et al., 2020; Ponomarenko et al., 2023).

### 3.4 Biological processes associated with the addition of lapatinib

We used Gene Ontology (GO) analysis to identify functional terms enriched among the detected DEPs. Each GO term identified is associated with a biological process affected by lapatinib, EGF, or human blood serum.

**FIGURE 3:**
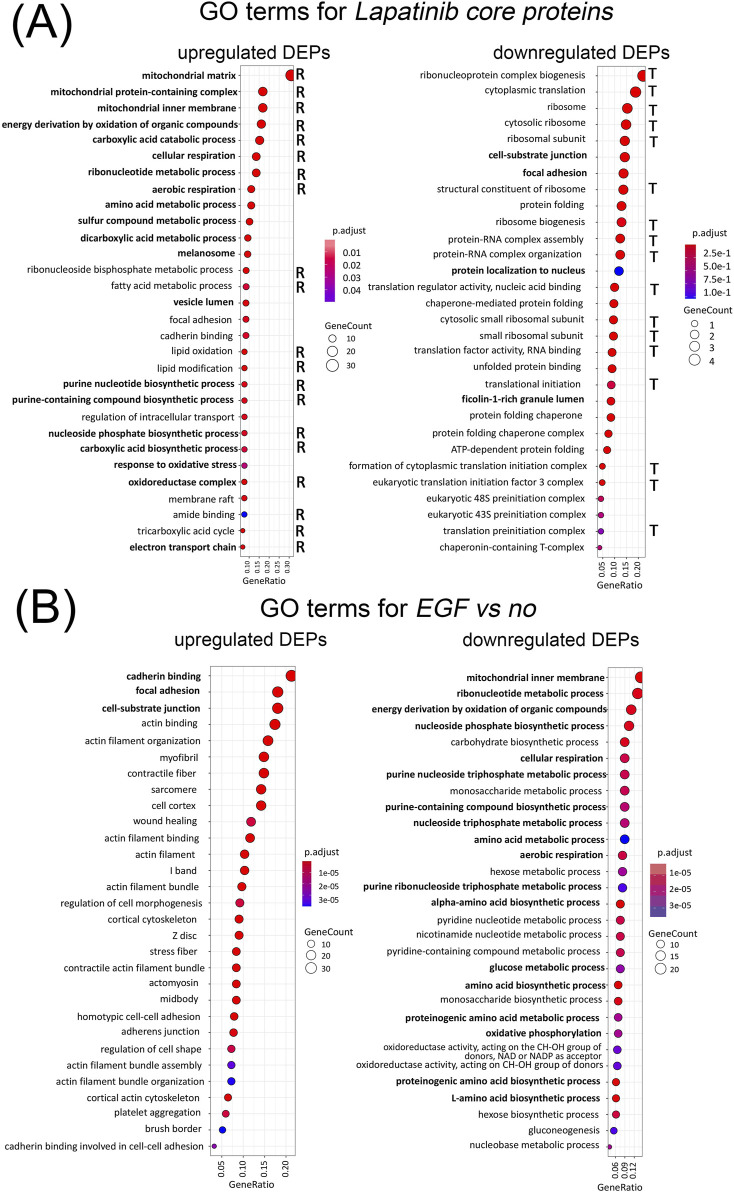
Gene Ontology terms enriched among Lapatinib core proteins with upregulated and downregulated expression as a result of lapatinib treatment. Terms relevant to cellular respiration are marked “R”, terms relevant to translation and ribosome function are marked “T” **(A)**. Gene Ontology terms enriched among proteins with upregulated and downregulated expression as a result of EGF treatment **(B)**. Terms that are common for lapatinib-induced DEPs and EGF-inhibited DEPs, and *vice versa*, are shown in bold. Enrichment of the indicated gene ontology terms is statistically significant (*p*-value adjusted by the Benjamin-Hochberg criteria, less than 0.001).

For the *Lapatinib core* protein set we identified 190 GO terms enriched among lapatinib-induced proteins and 375 GO terms enriched among lapatinib-inhibited proteins, Supplementary Table S2. GO terms have a hierarchy: some of them are generic and describe many biological processes, while others are more specialized and refer to a specific biological reaction. In such terms, many proteins overlap (Supplementary Table S2). To represent the 30 most strongly enriched GO terms in Figure 3, we combined synonymous terms.

Among top-30 GO-terms enriched in lapatinib core upregulated proteins, the majority (20 GO-terms) are related to mitochondrial membrane functioning, cellular respiration, oxidative phosphorylation and tricarboxylic acid cycle (TCA). Thus, we conclude that lapatinib activates processes dealing with cellular respiration. This corresponds to our observation of 41% of the *lapatinib* core proteins associated with mitochondrial function. Two other top GO terms are related to amino-acid metabolism, which products can also contribute to TCA. Among GO terms enriched with *lapatinib core* DEPs, some may be associated with cell proliferation arrest.

Addition of EGF or human serum together with lapatinib returns the protein levels and the associated pathways close to the non-drug level (Figure 2).

Totally, we detected 21 protein related to mitochondria which expression is altered by EGF treatment, all of them are downregulated. Among the GO terms enriched in lapatinib-induced DEPs, 75 GO terms (39%) are also enriched in EGF-inhibited DEPs (Supplementary Table S2). At the same time, only 8 proteins (2.6% of the lapatinib core DEPs) are shared between the lapatinib and EGF DEPs (Supplementary Table S1). Thus, the analysis of GO-enrichment of DEP sets provides a more comprehensive understanding of the alterations in cellular processes resulting from drug action compared to individual DEP comparisons.

Interestingly, among the top-30 cellular processes inhibited by EGF, 18 mainly related to cellular respiration are in contrast activated by lapatinib (Figure 3, bold).

Thus, EGF largely acts opposite to lapatinib on the proteome level. This also corresponds to the antagonistic functional activities of EGF and the EGFR/HER2 inhibitor lapatinib.

Among the GO terms enriched among the downregulated lapatinib core proteins, the majority related to translation and ribosome (17 out of 30); 7 GO terms deal with protein folding and chaperone functions (Figure 3). Previous studies employing alternative methods have also detected that lapatinib induces inhibition of translation (Adjibade et al., 2020). Among the remaining GO terms, some are related to cell membrane function and cell adhesion. This is consistent with the results of transcriptomic profiling of these cells under the same conditions reported previously by our group, where focal adhesion genes were strongly altered by lapatinib treatment (Shaban et al., 2024).

### 3.5 Analysis of intracellular molecular pathways associated with the effects of lapatinib, human blood serum and EGF

**FIGURE 4:**
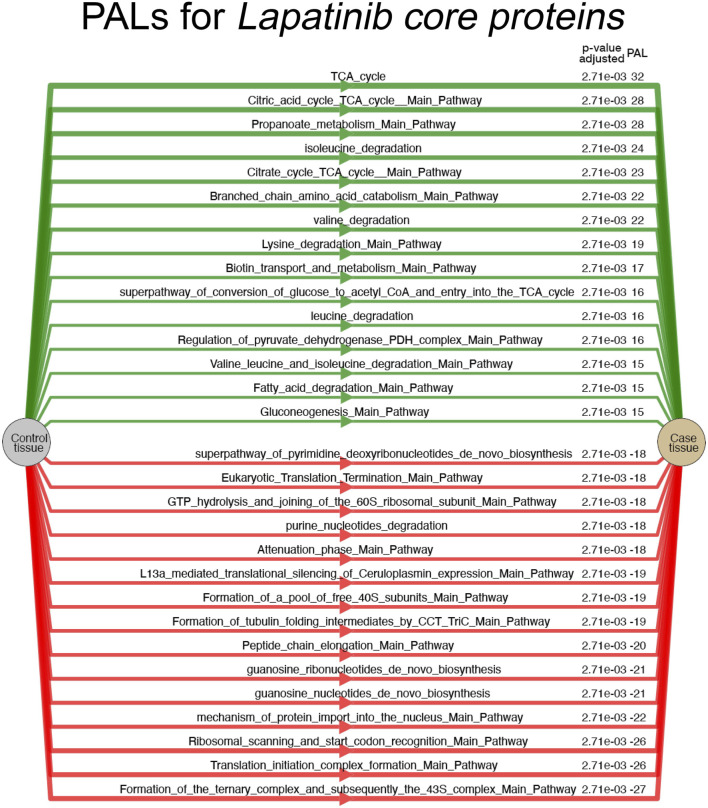
Top-30 most strongly activated and inhibited molecular pathways identified for a set of DEPs specific for lapatinib core signature in SK-BR-3 cells. PAL values and FDR-corrected *p*-values are shown for the respective pathways. PAL values and FDR-corrected *p*-values are shown for the respective pathways.

Proteomic results provide a list of DEPs under the impact of lapatinib, EGF, human serum and their combinations compared to drug-free controls. Using the online pathway analysis tool OncoboxPD, we calculated pathway activation levels (PALs) for 1,529 human intracellular molecular pathways with more than 10 participants. Differentially regulated pathways are summarized in Supplementary Table S3 for all five combinations of lapatinib, human serum and EGF compared to control cells. Top-30 pathways most strongly altered by the action of lapatinib are shown in Figure 4.

In this analysis, lapatinib treatment most significantly activated tricarboxylic acid cycle (TCA) pathways and inhibited translation-related pathways (Figure 4) which agrees with the previous GO analysis.

Thus, lapatinib treatment activates processes promoting the cellular respiration. Propanoate metabolism pathway links propanoil CoA production through the catabolism of specific amino acids or the oxidation of odd-chain fatty acids and links this catabolism to TCA. Conversion of glucose to Acetyl CoA super pathway is also among the most activated items. Coenzyme CoA links it to the TCA (Czumaj et al., 2020). Totally, 7 of 15 most activated by lapatinib treatment pathways relate to TCA. Six others, in turn, deal with the degradation of amino acids valine, leucine, isoleucine, and lysine. Ultimately, these amino acids are broken down into acetyl or propionyl CoA to enter the TCA cycle (Duarte et al., 2019). At the same time, combined treatment with lapatinib and human blood serum or EGF cancels activation of all aforementioned pathways (Supplementary Table S3).

We note that *Trafficking_and_processing_of_endosomal_TLR_Main_Pathway* is also activated by lapatinib treatment (PAL = 14.6, Supplementary Table S3) and not activated by combined treatment with lapatinib and EGF or lapatinib and human blood serum. This pathway alteration was found previously in the same conditions by our transcriptomic assay (Shaban et al., 2024).

The treatment with EGF only inhibits two out of seven TCA pathways shown in Figure 4, without affecting amino acid degradation pathways. Interestingly, Gluconeogenesis pathway that is activated by lapatinib, experiences the highest inhibition with EGF treatment (expression of eight proteins in this pathway is inhibited by EGF).

Lapatinib treatment most significantly inhibits pathways related to translation and protein folding, and also the pathways related to nucleotide biosynthesis (Figure 4).

**FIGURE 5:**
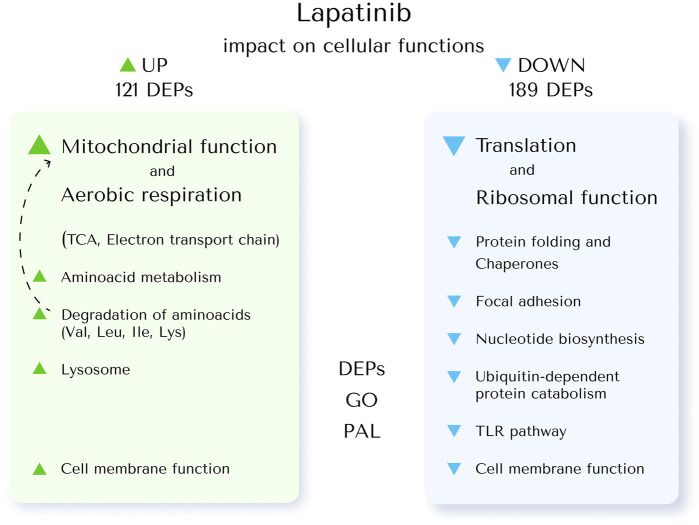
The impact of lapatinib on cellular functions revealed by proteome profiling. To investigate this, the core proteins upregulated (UP) and downregulated (DOWN) by lapatinib treatment in SK-BR-3 cells were associated with specific cellular functions (DEPs). GO terms enriched in these DEPs were analyzed (GO); pathway activation levels were calculated (PAL).

Summary of the lapatinib impact on cellular functions revealed by the proteme profiling is shown in the Figure 5.

**FIGURE 6:**
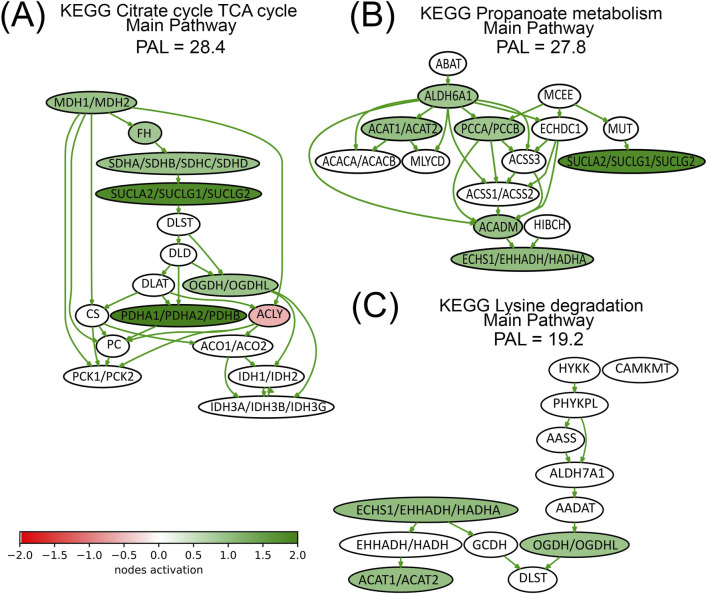
Activation charts for top-three lapatinib activated pathways (Panels **(A–C)**) shown as interacting networks. Pathway component activation is compared with no-drug conditions. Pathway activation level (PAL) is indicated for lapatinib treatment condition. Green/red arrows indicate activation/inhibition interactions, respectively. The color depth of nodes reflects the extent of node activation (natural logarithms of the expression fold change for each node, the reference is the geometric average between expression levels in all samples in the respective groups). Green stands for activation, red stands for inhibition, white stands for non-differential expression or no data available.

We present here the pathway activation charts of the three most strongly activated pathways calculated using the experimental proteomic data (Figure 6). The major components of *TCA pathway* (Figure 6A), are upregulated by lapatinib treatment. For example, lapatinib induced both proteins that constitute MDH1/MDH2 node (Table 2), as well as three out of four proteins that constitute the SDHA/SDHB/SDHC/SDHD node. Interestingly, one of the nodes, ACLY, is inhibited by lapatinib.

Three nodes from Lysine degradation pathway are upregulated by lapatinib (Figure 6C), and some of the proteins that constitute these nodes, HADHA, ACAT1, and OGDH, are associated with respiration and mitochondrial function (Table 2). Other pathway activation charts are presented in Supplementary Figure S1.

### 3.6 Lapatinib treatment influences the aerobic respiration in SK-BR-3 cells

Proteomic profiling revealed that the expression of multiple proteins associated with mitochondrial function, oxidative phosphorylation, and metabolic pathways linked to TCA is upregulated upon lapatinib treatment. Thus, we investigated the impact of lapatinib on cellular redox potential through the resazurin test. This assay, which involves the conversion of resazurin into fluorescent resorufin by NAD(P)H, is a well-established method for assessing mitochondrial respiration in mammalian cells (Lavogina et al., 2022).

**FIGURE 7:**
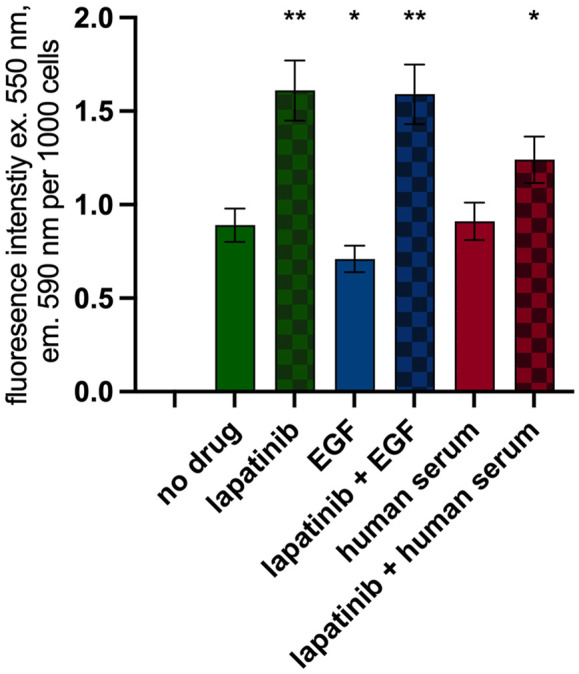
Resazurin test. Resorufin fluorescence normalized per 1,000 cells. Data (mean ± SEM) from three or more independent experiments are presented. ^*^ *p* < 0.05, ^**^ *p* < 0.01.

Lapatinib (150 nM), EGF (2 ng/mL) or 5% human serum was added to seeded SK-BR-3 cells. Then, after 48 h, cells were counted and the resazurin test was performed (Supplementary Figure S2). Due to the growth-inhibiting effects of lapatinib, the cell counts were markedly lower in lapatinib containing wells compared to the control group. Furthermore, we quantified resazurin fluorescence per thousand cells (Figure 7). Our findings revealed a 1.8-fold increase in NAD(P)H concentration following exposure to lapatinib. EGF treatment led to an 82% level of NAD(P)H compared to the drug-free level. Treatment with human serum showed no significant change in NAD(P)H concentration. Thus, the resazurin test confirms the enhancement of processes related to aerobic respiration of metabolically active cells induced by lapatinib, as well as mild attenuation of cellular respiration in response to EGF.

## 4 Discussion

Investigating the mechanisms of tumor resistance to drug therapy and developing strategies to overcome this resistance are key directions in cancer research. Previously, using HER2+ breast adenocarcinoma cell line SK-BR-3, we observed a noteworthy reduction in the inhibitory effects of the EGFR/HER2-targeting drug lapatinib on cell growth when human blood serum was present in the growth media (Shaban et al., 2024). Similarly, in our recent research involving the EGFR-positive A431 cell line, we have demonstrated a substantial increase of 5–25 times in the effective concentrations of the EGFR-targeting drugs cetuximab and erlotinib when human serum was added (Kamashev et al., 2023).

Thus, the presence of human serum can significantly alter the efficacy of HER-targeted drugs. Consequently, it is important to consider the potential effect of human peripheral blood on drug potency when evaluating the antitumor activity of experimental drugs in preclinical trials. In this study, we tested whether animal serum could have a similar effect on the efficacy of lapatinib, or whether the effect of serum on drug potency is species specific. It turned out that serum from horse, pig, goat, and cattle had no significant effect on the inhibition of cell growth by lapatinib and could not serve as a substitute for human serum in tests of drug efficacy.

In a previous study, we demonstrated a significant reduction in the inhibitory effect of lapatinib on cell growth in the presence of recombinant EGF at a concentration of 0.82–20 ng/mL in the medium (Shaban et al., 2024). To investigate whether endogenous EGF in human serum samples affects the efficacy of lapatinib, we quantified the level of EGF in human serum samples, which ranged from 0.6 to 0.8 ng/mL depending on the blood donor. Consequently, the medium used in our experiments (Figure 1) containing 5% serum contained approximately 0.035 ng/mL of EGF. Here we show that at this concentration rhEGF did not affect the efficacy of lapatinib. We conclude that the concentration of EGF in human serum is most probably too low to be responsible for the effect of serum on the efficacy of lapatinib (Figure 1).

We have previously shown that the effect of human serum on lapatinib efficacy is due to abrogation of the drug-induced arrest of the G1/S cell cycle transition. RNA sequencing showed that in combination with lapatinib, human serum or EGF could restore both cell growth rate and expression of approximately 96% of the genes that were altered by lapatinib treatment alone (Shaban et al., 2024). To further investigate the mechanism of action of lapatinib on cell growth and the interference of human serum and EGF in the action of lapatinib, we performed proteomic profiling of SK-BR-3 cells under the influence of lapatinib, EGF and human serum.

Proteomic profiling revealed 350 DEPs under the effect of lapatinib. Remarkably, ∼93% of these proteins ceased to be differentially expressed when human serum was added together with lapatinib. Thus, human serum not only restored the cell growth rate when lapatinib was added, but also restored the expression of lapatinib-specific DEPs. A similar pattern was observed when EGF was added together with lapatinib: only ∼7% (23 proteins) of lapatinib-specific DEPs remained differentially expressed. Proteins whose expression is altered by lapatinib treatment and not altered by combined treatment with lapatinib and human serum or EGF were named here as *lapatinib core proteins*. Among the others, these proteins may be responsible for the arrest of cell growth.

Our analysis revealed that lapatinib treatment most significantly inhibits pathways related to translation and protein folding, consistent with previous studies (Adjibade et al., 2020; Mitchell et al., 2010). We noted that lapatinib treatment reduces the expression of G3BP protein, which is involved in the mechanism of stress granule (SG) translation (Adjibade et al., 2020).

Analysis of GO terms enriched in lapatinib upregulated proteins demonstrates that the activation of cellular respiration by lapatinib is associated with cellular proliferation arrest (Figure 3). Based on proteome profiling data we calculated pathway activation levels (Figure 4). In a complete agreement with GO terms analysis we conclude that lapatinib treatment most significantly activates TCA cycle.

Propanoate metabolism pathway is also among the most activated by lapatinib treatment pathway. Propanoate metabolism depicts the metabolism of propionic acid. Propionyl-CoA cannot directly enter the beta-oxidation cycle so it is carboxylated into D-methylmalonyl-CoA and further converted to succinyl-CoA, an intermediate of the TCA cycle (MacFabe et al., 2007). The branched chain amino acids, leucine, isoleucine, and valine, can enter the mitochondrion as such via a neutral amino acid carrier protein, and are then converted into the 2-oxo-acids by a mitochondrial aminotransferase. Branched-chain 2-oxo-acids generated from leucine, isoleucine, and valine, then undergo oxidative decarboxylation by the branched-chain 2-oxo-acid dehydrogenase complex (BCKADH) to form the branched-chain acyl-CoA esters. The degradation of valine, leucine and isoleucine leads to the production of acetyl-CoA and propionyl-CoA. The propionyl-CoA is converted to the TCA intermediate succinyl-CoA. Thus, these amino acids can be used to synthesize glucose and other carbohydrates (Duarte et al., 2019).

Through the resazurin test, we showed that cellular respiration activity was increased 1.8-fold by lapatinib. The linkage between the arrest of SK-BR-3 cell proliferation and the enhancement of cellular respiration under lapatinib treatment is currently poorly understood. However, it would be intriguing to modify cellular respiration using specific drugs or hypoxia in combination with lapatinib in order to investigate the combined treatment effects on cell growth. For example, administering carnitine or mildonium during lapatinib treatment could markedly affect the efficacy of the drug in SK-BR-3 cells (Sjakste et al., 2005). L-carnitine enhances the transportation of fatty acids to mitochondria and augments cellular metabolic processes in the presence of oxygen, whereas mildonium serves as an endogenous carnitine antagonist (Pekala et al., 2011). Our research indicates that these substances have the potential to modify the impact of lapatinib on cells. Moreover, enriching the growth media with TCA intermediates like succinate could potentially alter the inhibitory influence of lapatinib. Additionally, hypoxia theoretically might also modulate lapatinib inhibitory effects on cell growth thus suggesting a new relevant direction of combined therapy research.

## Data Availability

The datasets presented in this study can be found in online repositories. The names of the repository/repositories and accession number(s) can be found below: http://www.proteomexchange.org/, PXD054180.
