# Supplementary material for: Lapatinib-induced enhancement of mitochondrial respiration in HER2-positive SK-BR-3 cells: mechanism revealed by analysis of proteomic but not transcriptomic data
Source: Front Mol Biosci. 2024 Sep 30;11:1470496. doi: 10.3389/fmolb.2024.1470496 (PMC11472020; doi:10.3389/fmolb.2024.1470496)
Supplement: Supplementary file 1 [file DataSheet1.ZIP › SKBR_suppementary_2024/Legends to supplementary Figures and Tables.docx]

Figure S1. Activation charts for two lapatinib core activated pathways (Panels A and B) is shown as an interacting network. Pathway components activation compared with no-drug conditions are shown. Pathway activation level (PAL) is indicated. Green/red arrows indicate activation/inhibition interactions, respectively. The color depth of nodes reflects the extent of node activation (natural logarithms of the expression fold change for each node, the reference is the geometric average between expression levels in all samples in the respective groups). Green stands for activation, red stands for inhibition, white stands for non-differential expression.

Figure S2. Resazurin test. Panel A: Dynamic range study for SK-BR-3 cells 24 h after seeding; the dashed line indicates the range in which the dependence of the assay readings on the number of cells seeded is linear. Panel B: Fluorescence of resarufin: in the absence of cells (no cells), in cells without drug (no drug), in the presence of 150 nM lapatinib, 2 ng/mL EGF, 5% human blood serum or their combitations. Panel C: Number of cells subjected to resazurin test. Panel D: Resarufin fluorescence normalized per 1000 cells. Data (mean ± SEM) from 3 or more independent experiments are presented. *p < 0.05, **p < 0.01.

Table S1. Obtained fold change (FC) values of DEPs (with criteria of FDR-adjusted p-value < 0.05; |log2(fold change)| >2). Columns: 1, Gene symbol; 2, Protein name; 4-12, protein functions; 13, lapatinib core; 14-23, log2FC and p-value adjusted for each of five treatments compared to no-drug control.

Table S2 List of GO terms enriched in upregulated (UP) and downregulated (DW) proteins compared with no-drug conditions for each treatment (Only GO terms with p-value adjusted < 0.05 are listed) // For lapatinib core up- and downregulated proteins detected GO terms are aligned with GO terms detected for other treatment conditions (if data are absent, this GO terms was not enriched significantly.

Table S3. Differentially regulated intracellular molecular pathways. Pathway names, Pathway activation values, p-values, and p-values adjusted are listed for each significantly altered compared to no drug conditions pathway.
