# Supplementary figures and images for: Lapatinib-induced enhancement of mitochondrial respiration in HER2-positive SK-BR-3 cells: mechanism revealed by analysis of proteomic but not transcriptomic data
Source: Front Mol Biosci. 2024 Sep 30;11:1470496. doi: 10.3389/fmolb.2024.1470496 (PMC11472020; doi:10.3389/fmolb.2024.1470496)

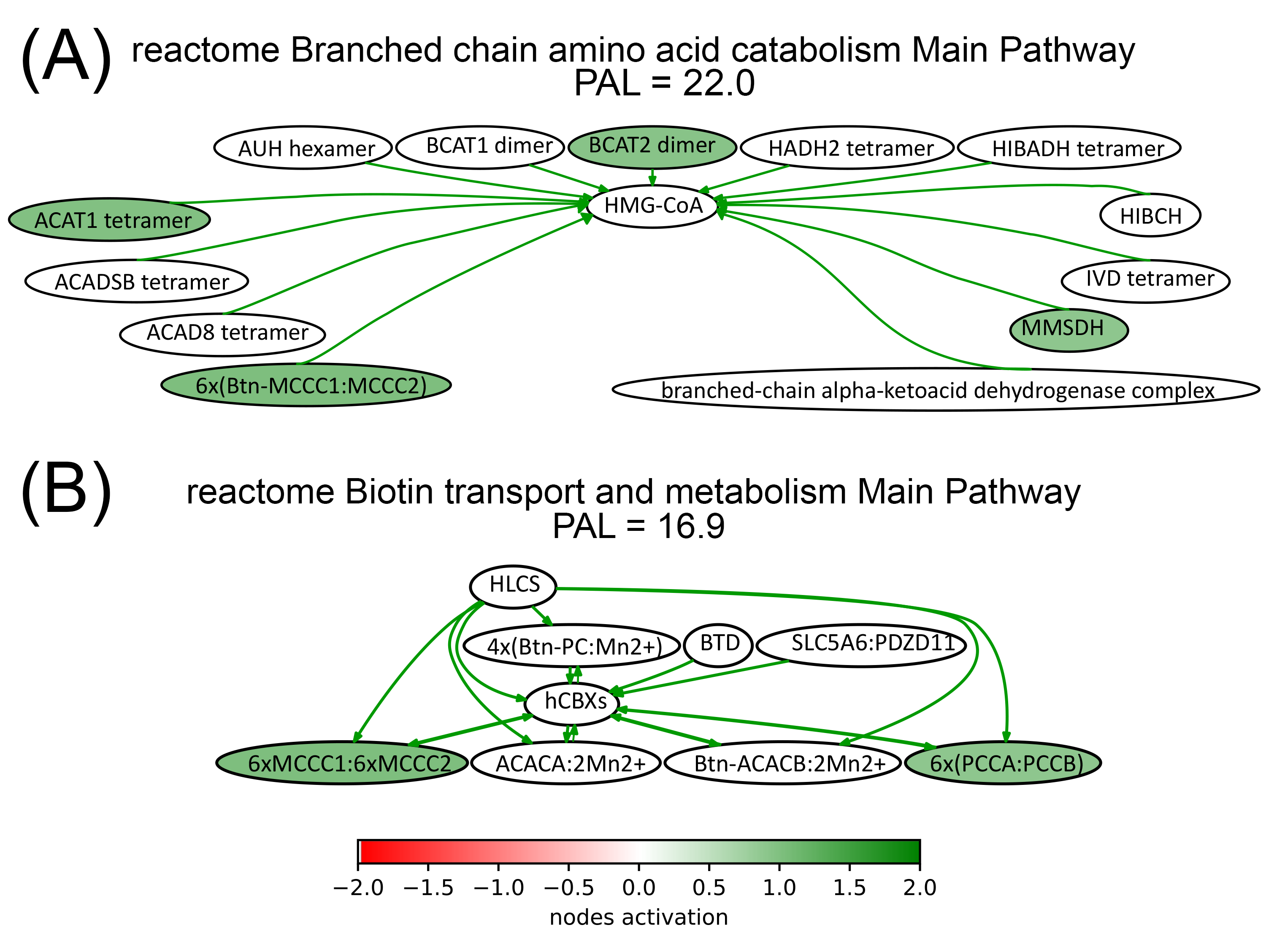

Supplement: Supplementary file 1 [file DataSheet1.ZIP › SKBR_suppementary_2024/figure S1.tif]

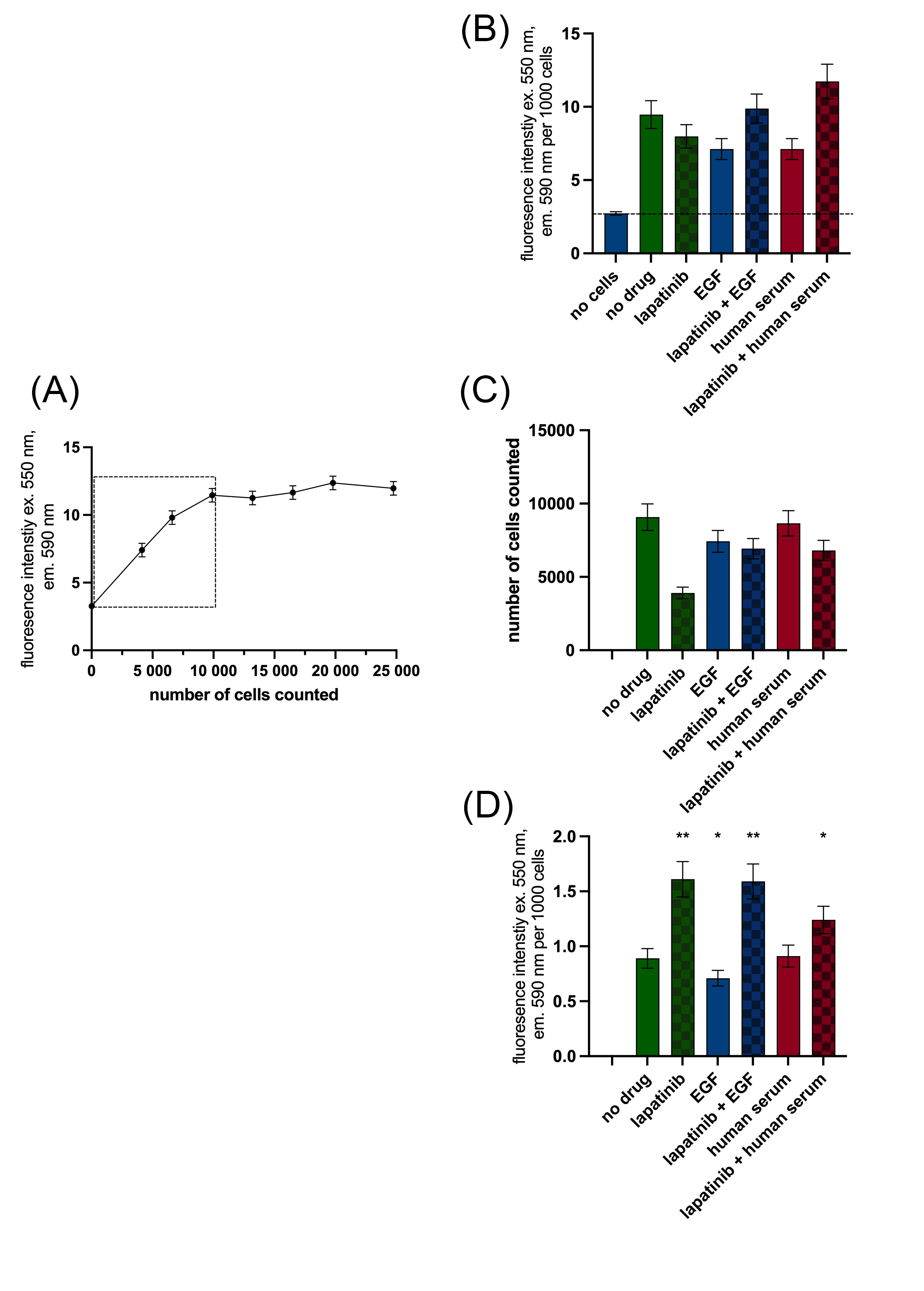

Supplement: Supplementary file 1 [file DataSheet1.ZIP › SKBR_suppementary_2024/figure S2.tif]
